# An improved prognostic model for predicting the mortality of critically ill patients: a retrospective cohort study

**DOI:** 10.1038/s41598-022-26086-1

**Published:** 2022-12-12

**Authors:** Xianming Zhang, Rui Yang, Yuanfei Tan, Yaoliang Zhou, Biyun Lu, Xiaoying Ji, Hongda Chen, Jinwen Cai

**Affiliations:** 1 Department of Respiratory and Critical Care Medicine, Affiliated Hospital of Guizhou Medical University, Guiyang City, Guizhou Province China; 2 Department of Endocrinology, Guiyang First People’s Hospital, Guiyang City, Guizhou Province China; 3 Department of Emergency, The Seventh Affiliated Hospital, Sun Yat-sen University, Shenzhen City, Guangdong Province China; 4 Department of Traditional Chinese Medicine, The Seventh Affiliated Hospital, Sun Yat-sen University, Shenzhen City, Guangdong Province China; 5 Department of Respiratory and Critical Care Medicine, The Third Xiangya Hospital of Central South University, Changsha City, Hunan Province China

**Keywords:** Medical research, Outcomes research

## Abstract

A simple prognostic model is needed for ICU patients. This study aimed to construct a modified prognostic model using easy-to-use indexes for prediction of the 28-day mortality of critically ill patients. Clinical information of ICU patients included in the Medical Information Mart for Intensive Care III (MIMIC-III) database were collected. After identifying independent risk factors for 28-day mortality, an improved mortality prediction model (mionl-MEWS) was constructed with multivariate logistic regression. We evaluated the predictive performance of mionl-MEWS using area under the receiver operating characteristic curve (AUROC), internal validation and fivefold cross validation. A nomogram was used for rapid calculation of predicted risks. A total of 51,121 patients were included with 34,081 patients in the development cohort and 17,040 patients in the validation cohort (17,040 patients). Six predictors, including Modified Early Warning Score, neutrophil-to-lymphocyte ratio, lactate, international normalized ratio, osmolarity level and metastatic cancer were integrated to construct the mionl-MEWS model with AUROC of 0.717 and 0.908 for the development and validation cohorts respectively. The mionl-MEWS model showed good validation capacities with clinical utility. The developed mionl-MEWS model yielded good predictive value for prediction of 28-day mortality in critically ill patients for assisting decision-making in ICU patients.

## Introduction

The health condition of intensive care unit (ICU) patients can vary radically depending on many factors, including previous health history, underestimation of illness severity, efficiency of care, and response to treatment^1^. Although great strides have been made in recent years in the field of critical care medicine, the mortality rate of ICU patients has seen only a small decrease and still remains around 20–40% due to the highly complex and heterogeneous diseases of these patients^2,3^.

In clinical practice, ICU prognostic models are critical for correctly evaluating and identifying high-risk ICU patients. This information helps clinicians to make appropriate medical judgements and prevent ICU deaths while also ensuring proper utilization of limited healthcare resources, especially in low- and middle-income countries (LMICs)^4–6^. These systems use bedside and digital distinguishing tools to identify the risk of serious aggravation and death in critical patients and can be used to help capture the intensity of resource utility and gain a better understanding of what constitutes true ICU-acquired organ dysfunction^7^. The predictive efficiencies of the commonly used scoring systems are reported in Table 1^8–10^. Among these systems, the Acute Physiology and Chronic Health Evaluation (APACHE)-II combines three critical domains to predict the mortality of patients: demographic features, such as age and sex; an evaluation of the patient’s chronic health status and admission diagnosis; and the worst values of 12 physiological variables during the first 24 h following ICU admission. However, use of the APACHE-II is time consuming and requires considerable medical expenses, because more than 20 clinical variables are needed to complete the scoring, which should be finished within 24 h after admission. Similar to the APACHE-II, a sequential organ failure assessment focusing on multiple-organ dysfunction is also inconvenient for rapidly assessing ICU patients. Therefore, it may be impractical to apply these scoring systems widely in resource-restricted settings as in LMICs^11^.

**Table 1:** Comparison of scoring systems for predicting ICU mortality.

Scoring systems	Patients	Mortality	ROC	Significance	Reference
APACHE II	2054 septic patients in ICU between June 2009 and February 2014	11.8%	0.80	APACHE II scores in septic patients were very strong predictors of hospital mortality	^8^
SAPS II	2470 cases of sepsis recorded in the MIMIC-III database from 2001 to 2012	20.4%	0.768	The scores of SOFA, SAPS-II, OASIS, and LODS can predict ICU mortality in patients with sepsis, but SAPS-II and OASIS scores have better predictive value than SOFA and LODS scores	^9^
SOFA			0.757		
OASIS			0.739		
**LODS**					
MEWS	292 shock patients	45.89%	0.614	Conventional MEWS but inferiority to the APACHE II	^10^

*APACHE II* Acute Physiologic Assessment and Chronic Health Evaluation II, *SOFA* Sequential Organ Failure Assessment, *SAPS-II* Simplified Acute Physiology Score II, *OASIS* Oxford Acute Severity of Illness Score, *LODS* Logistic Organ Dysfunction System, *MEWS* Modified Early Warning System.

The Modified Early Warning Score (MEWS) is a simple and efficient track-and-trigger system for identifying patients with acute illness. It is derived from five common and vital physiological signs: respiratory rate, body temperature, systolic blood pressure, pulse rate, and level of consciousness. This score is helpful for predicting ICU admission and in-hospital mortality through the detection of physiological abnormalities^12^. The MEWS has advantages in application as it uses easily measurable and available parameters, does not increase the burden of disease, and is suitable for resource-limited settings. In the Surviving Sepsis campaign guidelines, the MEWS is recommended as a screening tool for identifying and managing critically ill patients^13^. However, the MEWS has been determined to be inferior to the APACHE II in terms of predictive efficacy for ICU mortality. A comparative study regarding the predictive efficacy for 28-day mortality in shock patients reported area under the receiver operating characteristic curve (AUROC) values of 0.785 for the APACHE-II and 0.614 for the MEWS^10^.

An ideal risk scoring system for critically ill patients should be easy to use, with accurate and informative performance as well as a low cost in order to improve the treatment of ICU patients. However, development of such a system due has been difficult due to the highly complex and heterogeneous diseases of ICU patients. Some convenient laboratory indexes such as the neutrophil-to-lymphocyte ratio (NLR), red cell distribution width (RDW), lactate (lac) concentration, and osmolarity have been widely applied for the prediction of ICU mortality in multiple patient populations in the past few years^14–17^. Some reports also have shown that the combination of a scoring system with simple laboratory indexes can improve the predictive efficacy of traditional scoring systems. For example, in a 28-day mortality analysis of 292 shock patients, an innovative MEWS based on the conventional MEWS, age, and transcutaneous oxygen saturation (AUROC = 0.696) was shown to be superior to the conventional MEWS (AUROC = 0.614; *p* < 0.05)^10^. Therefore, the integration of a traditional scoring system and simple laboratory indexes might offer a scoring system with a high predictive efficacy suitable for utility in LMICs.

In this study, we developed an improved MEWS scoring system using convenient data, including the MEWS, NLR, lac concentration, international normalized ratio (INR), osmolarity level, and presence of metastatic cancer, by analyzing the correlation of each variable with 28-day ICU mortality. We then compared the predictive efficacies of different scoring systems for 28-day mortality in a development group and verified our developed model in a validation group using clinical data of ICU patients included in the Medical Information Mart for Intensive Care III (MIMIC-III) database.

## Methods

### Study design

This study analyzed a retrospective cohort of patients admitted to the ICU (aged 14 years or older). A new MEWS scoring system was developed with the aim of better predicting the 28-day all-cause mortality of critically ill patients with a validation display in a nomogram. The datasets used in this study were derived from the publicly available database MIMIC-III (version 1.4), which contains high-quality health-related data from patients who were admitted to the ICU of the Beth Israel Deaconess Medical Center between 2001 and 2012. After completing the National Institutes of Health web-based training course, we obtained approval to access the database (Certification Number: 37764466). Informed consent was not required because all protected health information had been de-identified.

### Study population

We reviewed the discharge summaries of all patients in the MIMIC-III database admitted to the ICU between 2001 and 2012. All ICU patients aged > 14 years old with a measured MEWS within 24 h after ICU admission were included in this study. Patients who met any of the following criteria were excluded: (1) age less than 14 years; (2) readmission in the same hospitalization (only data from the first ICU admission were included in forming the final cohort); (3) unavailability of MEWS due to omission of a measurement within 24 h after ICU admission. The screened ICU patients were eligible for subsequent analysis.

### Data extraction, management, and processing

Demographic, clinical, and laboratory data and risk scoring results were extracted with structured query language using PostgreSQL tools (version 9.6) or calculated from the following tables: ADMISSIONS, ICUSTAYS, CHARTEVENTS, DIAGNOSIS_ICD, d_items, d_icd_diagnoses, LABEVENTS, PATIENTS, prescriptions, and Materialized Views. The extracted items for demographic, clinical, and laboratory data and risk scoring results in the database are listed in Table 2. The data processing, including missing data imputation and Winsorizing, was only performed on the development set, and the validation set was used to validate the predictive performance of the developed model. The worst values for lab parameters were selected if they were measured multiple times within 48 h before and after ICU admission. The body mass index (BMI) was calculated as weight (kg)/height (m)^2^, and osmolarity was calculated as (2 × sodium + potassium) + (glucose/18) + (blood urea nitrogen/2.8). The risk scoring systems including the APACHE-II and MEWS. The APACHE II scoring system is based on 12 physiological variables (temperature, mean arterial pressure, heart rate, respiratory rate, alveolar-arterial oxygen tension difference [fraction of inspired oxygen (FiO_2_ > 50%)] or partial pressure of oxygen in the arterial blood (PaO_2_; FiO_2_ < 50%), arterial pH or HCO_3_, serum sodium, serum potassium, serum creatinine, hematocrit, white blood cell count, and the Glasgow Coma Scale score, a chronic health evaluation and age adjustment score. Each variable was calculated using the worst values for these parameters recorded during the first 24 h in the ICU; the range for the total Apache II score is 0–71 points. The APACHE-II scores for all ICU patients were acquired with the SQL code from the Materialized Views of the MIMIC-III database. The MEWS was calculated according to Table 3^7^ with the worst values within 24 h after ICU admission selected for the parameter used for the MEWS evaluation.

**Table 2:** Demographic, clinical, laboratory and risk scoring systems extracted from the database.

Demographic information	Clinical characteristics (preexisting chronic medical conditions or comorbidities)	Laboratory parameters		Risk scoring systems	
Age	Congestive heart failure	Other neurological abnormality	White blood cell counts (WBC)	International normalized ratio (Inr)	Apache II
Gender	Cardiac arrhythmias	Psychoses	Neutrophil-to-lymphocyte ratio (NLR)	Ph	
BMI	Pulmonary circulation abnormality	Depression	Hemoglobin (HGB)	Pao_2_	
Admission date into ICU	Valvular disease	Solid tumor	Red cell distribution width (RDW),	PaCO_2_	
Discharge date out of ICU	Peripheral vascular disease	Metastatic cancer	Platelets	SO_2_	
Date of death	Hypertension	Lymphoma		PaO_2_/FiO_2_	
	Application of vasoactive drug used within 48 h before and after ICU admission)	Coagulopathy	Total bilirubin (TBIL)	Lactate	
	Chronic pulmonary disease	Blood loss anemia	Aspartate transaminase (AST),	Sodium	
	Liver disease	Deficiency anemias	Alanine Transaminase (ALT)	Potassium	
	Peptic ulcer	Alcohol abuse	Blood glucose (GLU)		
	Renal failure	Drug abuse	Blood urea nitrogen (BUN)		
	Diabetes	Rheumatoid arthritis	Serum creatinine (SCR)		
	Hypothyroidism	Acquired immune deficiency syndrome	Prothrombin time (PT),		
	Paralysis		Activated partial thromboplastin time (APTT)

**Table 3:** Details of modified early warning score.

Score	3	2	1	0	1	2	3
Respiratory rate (min^−1^)		≤ 9		9–14	15–20	21–29	≥ 30
Heart rate (min^−1^)		≤ 40	41–50	51–100	101–110	111–129	≥ 130
Systolic BP (mmHg)	< 70	71–80	81–100	101–199		≥ 200	
Temperature (°C)		< 35		35–38.4		> 38.5	
Neurological				Alert	Reacting to voice	Reacting to pain	Unresponsive

Because the true ages of patients over 89 years old were omitted due to the privacy policy of the MIMIC database, we selected age × 90/300 as a surrogate age for those patients. In data processing, we used multiple imputation to fulfill missing values based on patients with known values that were most similar to those patients with missing values. The missing data were predicted by the relationship between variables, and multiple complete datasets were generated by the Monte Carlo method. After analyzing these datasets, the analysis results were summarized. After imputations, we selected Winsor means to duplicate outliers with the command of winsor2 with replace cuts (1,99). To further examine the effect of osmolarity, the data were additionally categorized into different levels for analysis in the logistic regression models, which could facilitate quick individualized scoring for further validation and clinical utility as follows: osmolarity level 1, < 300 mmoL/L; level 2, ≥ 300 mmoL/L.

### Development of the risk prediction model and model validation

The eligible patients were randomly assigned at a ratio of 2:1 to either the derivation cohort for model development or the internal validation cohort for model verification. We performed an initial analysis of all available variables between survivors and nonsurvivors in the development and validation cohorts. Univariate and multivariate logistic analyses were used to identify independent predictors for 28-day all-cause mortality of critically ill patients and to develop the predictive model. Collinearity analysis was used to avoid potential multicollinearity of the predictive model. The discriminative performance of the obtained predictive model was compared with that of the APACHE-II, MEWS, RDW, NLR, lac, and osmolarity in the development and validation groups based on AUROC and 95% confidence interval (CI) values. Calibration of the constructed model was assessed by the H/L C-statistic and calibration curves, and the accuracy of the constructed model was evaluated by the Brier score. Precision-recall area under the curve (PR-AUC) values were calculated for the constructed model and the APACHE-II using the validation cohort.

### k-Fold cross validation of the mionl-MEWS score

We performed k-fold cross-validation with five random folds for the total of 51,121 patients. We compared the AUROC, positive predictive value (PPV), and negative predictive value (NPV) values between the model and cross-validation to show the robustness of our model.

### Nomogram development for the simplified prediction model

A nomogram is a graphical tool that can be easily used by clinicians in a resource-limited environment, as no statistical software or online electronic calculator is required. In this study, a nomogram was formulated with clinical practicability based on the results for the obtained predictive model.

### Statistical analysis

All patients were divided into two cohorts (development vs. validation) with complete randomization. The distributions of continuous variables were assessed by the Kolmogorov–Smirnov test, and data with skewed distributions were log normalized. Normally distributed continuous variables were expressed as mean ± standard deviation (SD), and non-normally distributed continuous variables were expressed as median (interquartile range). Categorical variables were expressed as absolute values (percentages). Descriptive statistics from the development and validation cohorts were used to compare the baseline data between survivors and nonsurvivors with the t test for normally distributed data, the Mann–Whitney U test for non-normally distributed data, and the chi-squared test for categorical variables. The covariates associated with 28-day all-cause mortality were further identified with univariate and multivariate logistic regression analyses. For each variable, the unadjusted and adjusted odds ratios (ORs) were assessed and reported with *p*-values and 95% CIs. The multivariate logistic regression model (mionl-MEWS) was built using a forward selection modeling process with a significance level of 0.05. The variables independently associated with 28-day mortality (metastatic cancer, MEWS, lac concentration, NLR, INR, and osmolarity level) were included in the final model. Furthermore, potential multicollinearity was tested using a mean variance inflation factor (VIF), where a value ≥ 10 indicated multicollinearity. Additionally, we assessed the discriminative abilities of the different models based on AUROC values. We then applied the obtained model generated from the development dataset to the validation dataset and assessed the discriminative ability based on the AUROC and the calibration capacity based on the H/L C-statistic. We also generated the calibration curves and calculated the Brier scores for predicting mortality among both the development and validation cohorts. The PR-AUC was applied to evaluate the predictive performance considering clinical application with the validation cohort. The robustness of the developed model was evaluated via k-fold cross validation. To enhance the clinical utility of the model, a nomogram was constructed based on the results of the multivariate analysis. All analyses were performed using Stata software (StataCorp. 2017, Stata Statistical Software: Release 15, College Station, TX: StataCorp LLC, version 14.0). A two-sided *p* < 0.05 was considered statistically significant.

### Ethical approval and consent to participate

Informed consent was not required because all protected health information had been de-identified.

## Results

### Study population and baseline characteristics

**Figure 1:**
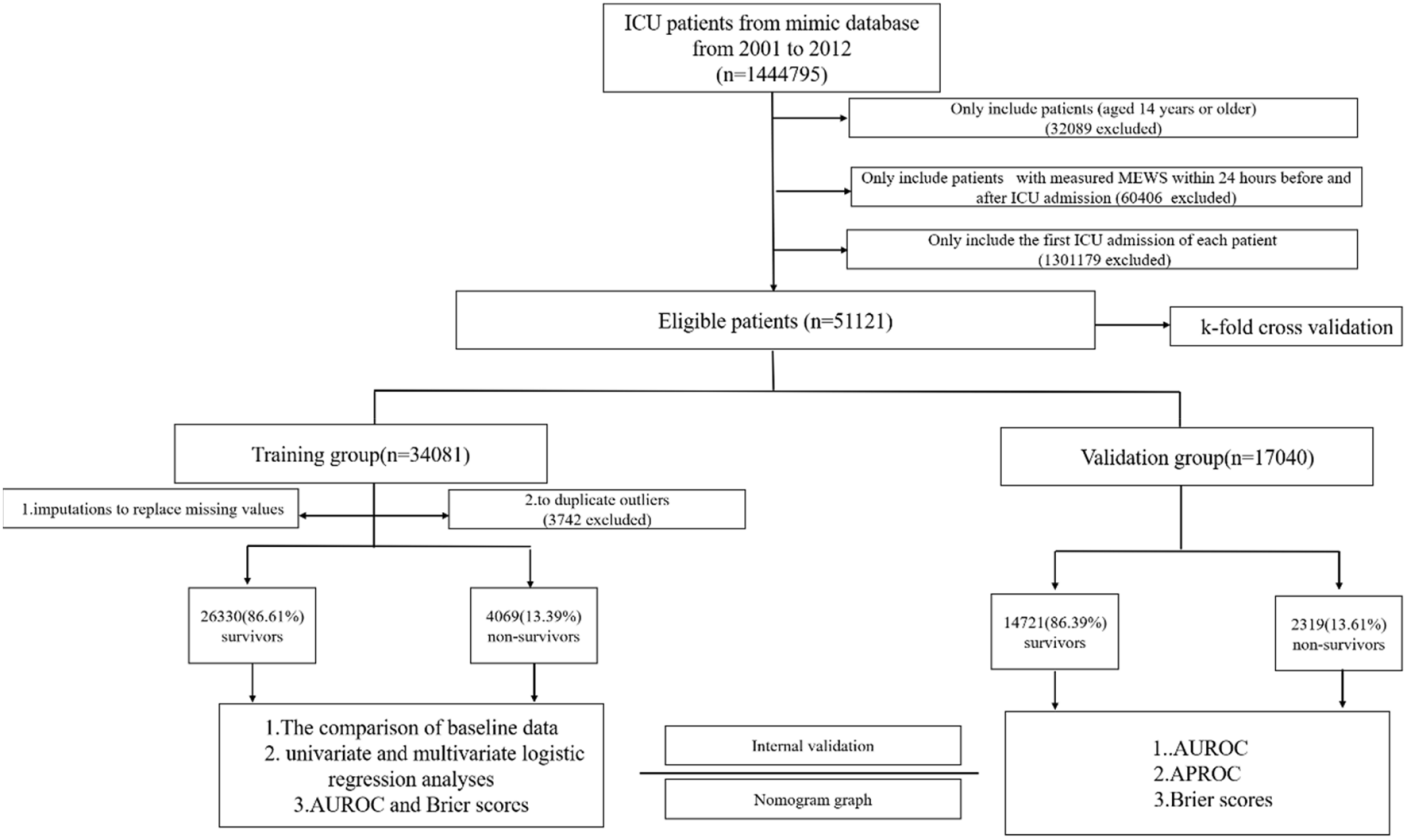
Study population and protocol flowchart. Flow chart illustrating the major steps in the development and validation of the mionl-MEWS model.

According to the inclusion and exclusion criteria, 1,444,795 ICU patients were selected from the MIMIC-III database. Of these, we excluded 1,301,179 cases as repeated ICU admissions, 32,089 patients with an age < 14 years, and 60,406 patients because the MEWS was not measured within 24 h before or after ICU admission. In total, 51,121 cases with sufficient data were included in the final analysis, including 28,742 male patients (55.52%) and 22,379 female patients (44.48%). The mean age of all patients was 74.80 ± 55.04 years. A total of 6825 patients died within 28 days, establishing an initial 28-day mortality rate of 13.35%. The detailed process of study population selection is shown in Fig. 1. Hypertension (54.68%) was the most common comorbidity, followed by cardiac arrhythmia (30.00%), diabetes (28.15%), and congestive heart failure (28.05%). In our study, 34,081 patients (66.67%) were randomly assigned to the development cohort, and 17,040 patients (33.33%) were assigned to the validation cohort.

### Development of a risk prediction model for 28-day all-cause mortality of ICU patients

The 28-day all-cause mortality percentages among critically ill patients were 13.39% in the development cohort (4069/30,399) and 13.61% in the validation cohort (2319/17,040). Significant differences in baseline clinical features, risk scores, and laboratory data were observed between survivors and nonsurvivors, as summarized in Tables 4 and 5. In the development cohort, nonsurvivors were predominantly male and compared with survivors, they had a significantly higher incidence of chronic medical conditions or comorbidities such as congestive heart failure, cardiac arrhythmia, pulmonary circulation disease, vasoactive drug use, liver disease, renal failure, hypothyroidism, paralysis, other neurological disease, solid tumor, metastatic cancer, lymphoma, and coagulopathy; and had a significantly lower incidence of valvular disease, hypertension, diabetes, psychoses, depression, and alcohol or drug abuse. Compared with survivors, nonsurvivors also were older and had significantly higher values for length of ICU stay, APACHE-II score, MEWS, white blood cell count, RDW, NLR, platelet (PLT) count, total bilirubin level, INR, aspartate transaminase level, alanine transaminase level, prothrombin time, activated partial thromboplastin time, blood urea nitrogen level, serum creatinine level, blood glucose level, lac concentration, osmolarity, and sodium level. In addition, the nonsurvivors had significantly lower hemoglobin, pH, PaO_2_, PaCO_2_, SO_2_, and PaO_2_/FiO_2_ values compared with the survivors. No significant differences in the rates of peripheral vascular disease, chronic pulmonary disease, peptic ulcer, anemia due to blood loss, deficiency anemia, rheumatoid arthritis, or acquired immune deficiency syndrome were observed between survivors and nonsurvivors in the development cohort. However, significant differences in the incidence rates like congestive heart failure, valvular disease, peripheral vascular disease, anemia due to blood loss, rheumatoid arthritis, and acquired immune deficiency syndrome etc. were also observed between the survivors and nonsurvivors in the validation cohort. The baseline characteristics showed similar distributions between the development and validation cohorts, indicating the successful randomization in the present study.

**Table 4:** Baseline characteristics and comparisons of demographics data, chronic medical conditions or comorbidities, risk scores and laboratory parameters of the study population between different survival status in development group.

	Survivors (n = 26,373)	Non-survivors (n = 4078)	*p*		Survivors (n = 26,373)	Non-survivors (n = 4078)	*p*
**Demographic data**				**Risk scores**			
Age (years)	64.67 (52.15–76.59)	74.74 (61.24–83.59)	< 0.001	APACHE-II	39 (29–51)	59(45–71)	< 0.001
Body mass index (kg/m^2^)	27.99 (23.52–34.85)	26.72 (22.26–33.65)	< 0.001	MEWS	5 (4–7)	7(5–9)	< 0.001
Sex, n (%)			< 0.001	63.14 ± 17.04	71.30 ± 15.32		
Female	11,484 (43.62%)	1904 (46.79%)		31.64 ± 14.61	30.64 ± 14.90		
Male	14,846 (56.38%)	2165 (53.21%)					
Length of ICU stay (days)	2.14 (1.22–4.14)	2.92 (1.46–6.16)	< 0.001				
**Chronic medical conditions or comorbidities**				**Laboratory parameters**			
Heart and great vessel disease				Blood routine examination			
Congestive heart failure	7320 (27.80%)	1506 (37.01%)	< 0.001	White blood cell counts (× 10^9^/L)	13.49 ± 5.67	16.44 ± 7.37	< 0.001
				Neutrophil to lymphocyte ratio	9.01 (5.52–12.73)	10.96(7.18–14.93)	< 0.001
Cardiac arrhythmias	7763 (29.48%)	1581 (38.85%)	< 0.001	Hemoglobin (g/L)	115.53 ± 20.68	110.41 ± 21.10	< 0.001
Pulmonary circulation disease	2041 (7.64%)	366 (8.99%)	0.006	Red cell distribution width (%)	15.06 ± 1.45	15.86 ± 1.63	< 0.001
Valvular disease	4164 (15.81%)	558 (13.71%)	0.001	Platelet count (× 10^12^/L)	250.49 ± 94.90	251.93 ± 112.54	< 0.001
Peripheral vascular disease	2762 (10.49%)	446 (10.96%)	0.363	Liver function test			
Hypertension	14,496 (56.95%)	2101 (51.63%)	< 0.001	Total bilirubin (mg/dL)	0.60 (0.40–0.90)	0.70(0.40–1.50)	< 0.001
Use of vasoactive drug (− 48 to 48 h)	4614 (17.52%)	1131 (27.80%)	< 0.001	Aspartate transaminase (U/L)	30.00 (21.00–59.00)	52.00(27.00–143.00)	< 0.001
Chronic pulmonary disease	5325 (20.22%)	853 (20.96%)	0.275	Alanine transaminase (U/L)	25.00 (16.00–44.00)	34.00(19.00–86.00)	< 0.001
Digestive system diseases				Coagulation function			
Liver disease	2058 (7.82%)	506 (12.44%)	< 0.001	International normalized ratio	1.60 (1.20–2.70)	1.90(1.30–3.10)	< 0.001
Peptic ulcer	30 (0.11%)	6 (0.15%)	0.563	Prothrombin time (s)	14.10 (13.00–15.80)	15.30(13.60–20.10)	< 0.001
Renal failure	4206 (15.97%)	811 (19.93%)	< 0.001	Activated partial thrombo-plastin time (s)	31.60 (27.00–43.80)	36.00(28.30–63.60)	< 0.001
Endocrine system diseases				Kidney function			
Diabetes	7610 (28.90%)	1111 (27.30%)	0.036	Blood urea nitrogen (mg/dL)	19.00 (14.00–28.00)	31.00(20.00–49.00)	< 0.001
Hypothyroidism	2740 (10.41%)	473 (11.62%)	0.019	Serum creatinine (mg/dL)	1.00 (0.80–1.30)	1.40(1.00–2.40)	< 0.001
Neurological and psychiatric diseases				Blood gas analysis			
Paralysis	927 (3.52%)	174 (4.28%)	0.016	PH	7.38 ± 0.08	7.36 ± 0.10	< 0.001
Other neurological disease	3165 (12.02%)	614 (15.09%)	< 0.001	PaO_2_ (mmHg)	168.86 ± 107.28	148.34 ± 98.53	< 0.001
Psychoses	1085 (4.12%)	101 (2.48%)	< 0.001	PaCO_2_ (mmHg)	41.56 ± 9.68	40.76 ± 11.91	< 0.001
Depression	2504 (9.51%)	242 (5.95%)	< 0.001	SO_2_ (%)	97.00 (95.00–98.00)	97.00(93.00–98.00)	0.002
Tumor				PaO_2_/FiO_2_	276.74 ± 119.51	248.43 ± 127.06	< 0.001
Solid tumor	1229 (4.67%)	257 (6.32%)	< 0.001	Lactate (mmol/L)	2.54 ± 1.43	3.32 ± 2.12	< 0.001
Metastatic cancer	1341 (5.09%)	537 (13.20%)	< 0.001	Electrolyte			
Lymphoma	488 (1.85%)	128 (3.15%)	< 0.001	Sodium (mmol/L)	139.64 ± 3.91	140.67 ± 5.52	< 0.001
Hematological diseases				Potassium (mmol/L)	4.81 ± 0.90	4.80 ± 0.95	0.501
Coagulopathy	2809 (10.67%)	817 (20.08%)	< 0.001	Osmolarity	305.28 ± 9.31	310.84 ± 12.66	< 0.001
Blood loss anemia	575 (2.18%)	82 (2.02%)	0.491	≥ 300 (mmoL/L)	71.35%	83.68%	< 0.001
Deficiency anemia	5129 (19.48%)	755 (18.55%)	= 0.165	Blood glucose (mg/dL)	162.03 ± 64.05	187.29 ± 82.07	< 0.001
Alcohol abuse	2025 (7.69%)	249 (6.12%)	< 0.001				< 0.001
Drug abuse	894 (3.40%)	70 (1.72%)	< 0.001				
Rheumatoid arthritis	810 (3.08%)	118 (2.90%)	0.543				
Acquired immune deficiency syndrome (AIDS)	292 (1.11%)	36 (0.88%)	0.198				

The normally-distributed continuous variables are shown as mean values and standard errors. The non-normally-distributed continuous variables are shown as medians. The categorical variables are shown as proportions of each subgroup. The comparison of baseline data between survivors and nonsurvivors is performed by t test for normally distributed data, the Mann–Whitney U test for non-normally distributed data, and the chi-squared test for categorical variables.

**Table 5:** Baseline characteristics and comparisons of demographics data, chronic medical conditions or comorbidities, risk scores and laboratory parameters of the study population between different survival status in validation group.

	Survivors (n = 14,721)	Non-survivors (n = 2319)	*p*		Survivors (n = 14,721)	Non-survivors (n = 2319)	*p*
**Demographic data**				**Risk scores**			
Age (years)	64.20 (51.29–76.55)	75.00 (61.92–83.62)	< 0.001	APACHE-II	38 (28–50)	58 (44–71)	< 0.001
Body mass index (kg/m^2^)	27.28 (23.71–31.87)	26.17 (22.41–30.81)	< 0.001	MEWS	5 (3–7)	7 (5–9)	< 0.001
Sex, n (%)			0.057				
Female	6292 (42.74%)	1040 (44.84%)					
Male	8429 (57.26%)	1279 (55.15%)					
Length of ICU stay (days)	2.09 (1.21–3.96)	2.84 (1.33–6.04)	< 0.001				
**Chronic medical conditions or comorbidities**				**Laboratory parameters**			
Heart and great vessel disease				Blood routine examination			
Congestive heart failure	3954 (26.86%)	825 (35.58%)	< 0.001	White blood cell counts (× 10^9^/L)	12.40 (9.40–16.20)	15.00 (10.80–20.00)	< 0.001
				Neutrophil to lymphocyte ratio	5.76 (3.32–10.39)	8.82 (5.15–16.73)	< 0.001
Cardiac arrhythmias	4298 (29.20%)	868 (37.43%)	< 0.001	Hemoglobin (g/L)	115.92 ± 26.84	111.01 ± 24.04	< 0.001
Pulmonary circulation disease	1032 (7.01%)	196 (8.45%)	0.013	Red cell distribution width (%)	14.62 ± 1.85	15.89 ± 2.38	< 0.001
				Platelet count (× 10^12^/L)	252.35 ± 107.02	249.65 ± 125.56	< 0.001
Valvular disease	2249 (15.28%)	307 (13.24%)	0.011	Liver function test			
Peripheral vascular disease	1595 (10.83%)	265 (11.43%)	0.395	Total bilirubin (mg/dL)	0.60 (0.40–0.90)	0.80 (040–1.60)	< 0.001
Hypertension	8117 (55.14%)	1287 (55.50%)	0.747	Aspartate transaminase (U/L)	31.00 (21.00–62.00)	54.00 (27.00–142.00)	< 0.001
Use of vasoactive drug (− 48 to 48 h)	2458 (16.70%)	618 (26.65%)	< 0.001	Alanine transaminase (U/L)	25.00 (16.00–46.00)	18.00 (34.00–95.00)	< 0.001
Chronic pulmonary disease	2942 (19.99%)	523 (22.55%)	0.004	Coagulation function			
Digestive system diseases				International normalized ratio	1.20 (1.10–1.50)	1.50 (1.20–2.20)	< 0.001
Liver disease	1199 (8.14%)	266 (11.47%)	< 0.001	Prothrombin time (s)	14.00 (13.00–15.60)	15.60 (13.70–19.90)	< 0.001
Peptic ulcer	28 (0.10%)	0 (0.00%)	0.036	Activated partial thrombo-plastin time (s)	31.20 (26.80–41.90)	35.35 (28.20–61.45)	< 0.001
Renal failure	2347 (15.94%)	436 (18.80%)	0.001	Kidney function			
Endocrine system diseases				Blood urea nitrogen (mg/dL)	19.00 (14.00–27.00)	30.00 (20.00–50.00)	< 0.001
Diabetes	4116 (27.96%)	634 (27.34%)	0.536	Serum creatinine (mg/dL)	1.00 (0.80–1.30)	1.35 (0.90–2.50)	< 0.001
Hypothyroidism	1432 (9.73%)	242 (10.44%)	0.287	Blood gas analysis			
Neurological and psychiatric diseases				PH	7.38 ± 0.08	7.35 ± 0.12	< 0.001
Paralysis	538 (3.65%)	125 (5.39%)	< 0.001	PaO_2_ (mmHg)	171.62 ± 113.06	149.52 ± 106.68	< 0.001
Other neurological disease	1756 (11.93%)	374 (16.13%)	< 0.001	PaCO_2_ (mmHg)	41.93 ± 11.14	42.20 ± 15.69	0.020
Psychoses	644 (4.37%)	68 (2.93%)	< 0.001	SO_2_ (%)	97.00 (95.00–98.00)	97.00 (93.00–98.00)	0.002
Depression	1398 (9.50%)	139 (5.99%)	< 0.001	PaO_2_/FiO_2_	279.15 ± 132.07	253.58 ± 146.87	< 0.001
Tumor				Lactate (mmol/L)	2.10 (1.40–3.20)	2.70 (1.60–5.60)	< 0.001
Solid tumor	665 (4.52%)	144 (6.21%)	< 0.001	Electrolyte			
Metastatic cancer	668 (4.54%)	306 (13.20%)	< 0.001	Sodium (mmol/L)	139.69 ± 4.44	141.20 ± 7.08	< 0.001
Lymphoma	285 (1.94%)	64 (2.76%)	0.009	Potassium (mmol/L)	4.80 ± 0.98	4.87 ± 1.11	= 0.092
Hematological diseases				Osmolarity	303.20 ± 10.82	316.55 ± 20.47	< 0.001
Coagulopathy	1566 (10.64%)	401 (17.29%)	< 0.001	≥ 300 (mmoL/L)	61.58%	81.23%	< 0.001
Blood loss anemia	317 (2.15%)	49 (2.11%)	0.901	Blood glucose (mg/dL)	168.04 ± 93.44	202.26 ± 112.64	< 0.001
Deficiency anemia	2795 (18.99%)	396 (17.08%)	0.028				
Alcohol abuse	1180 (8.02%)	152 (6.55%)	0.015				
Drug abuse	586 (3.98%)	35 (1.51%)	< 0.001				
Rheumatoid arthritis	444 (3.02%)	75 (3.23%)	0.570				
Acquired immune deficiency syndrome (AIDS)	156 (1.06%)	28 (1.21%)	0.522				

**Figure 2:**
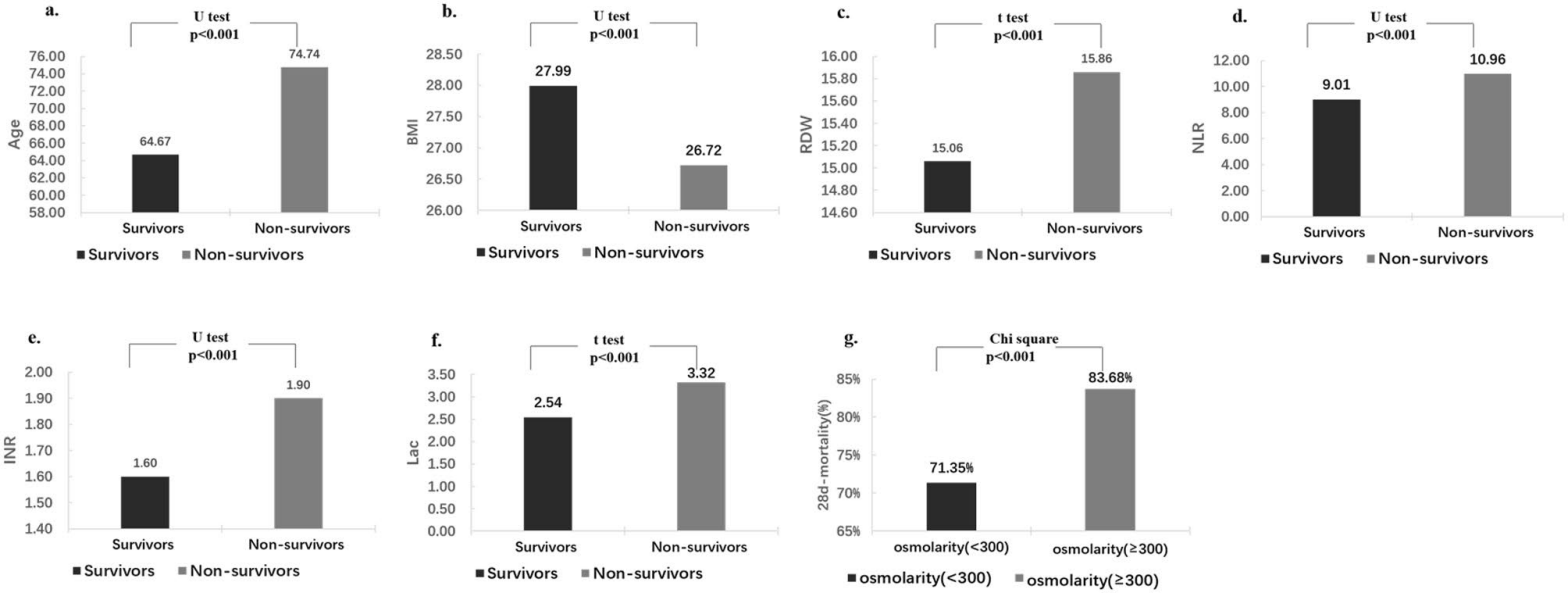
Comparisons of age, body mass index (BMI), red cell distribution width (RDW), neutrophil-to-lymphocyte ratio (NLR), international normalized ratio (INR), lactate (lac) concentration, and osmolarity between survivors and nonsurvivors in the development cohort. (**a**) Comparison of age by the U test, *p* < 0.001; (**b**) comparison of BMI by the U test, *p* < 0.001; (**c**) comparison of RDW by the t test, *p* < 0.001; (**d**) comparison of NLR by the U test, *p* < 0.001; (**e**) comparison of INR by the U test, *p* < 0.001; (**f**) comparison of lac concentration by the t test, *p* < 0.001; and (**g**) comparison of osmolarity by the chi-squared test, *p* < 0.001.

Next, we included the variables that differed significantly between survivors and nonsurvivors of the development cohort in univariate logistic regression analysis. The results presented in Table 6 demonstrated that all selected variables were significantly associated with 28-day mortality in the univariate logistic regression analysis, similar to the results of the abovementioned univariate analyses. The demographic characteristics with the three largest OR values were: age, OR = 1.033, *p* < 0.001; BMI, OR = 0.995, *p* < 0.001; and sex, OR = 0.880, *p* < 0.001. The three chronic medical conditions or comorbidities with the largest OR values were: metastatic cancer, OR = 2.833, *p* < 0.001; coagulopathy, OR = 2.100, *p* < 0.001; and requirement of vasoactive drug therapy, OR = 1.812, *p* < 0.001. For risk scores and laboratory parameters, we selected the indicators with a low cost and a high frequency of use in the ICU. For example, the MEWS can be obtained by simple calculation with the parameters on the nursing record sheet; the RDW and NLR can be obtained via routine blood tests; and the lac concentration, INR, and osmolarity can be obtained using portable testing tools. Regarding the lac concentration, INR, RDW, NLR, and osmolarity, significantly increasing 28-day mortality rates were observed in patients with a lower BMI or a higher age, INR, RDW, and osmolarity level (osmolarity ≥ 300: nonsurvivors, 83.68% vs. survivors, 71.35%, *p* < 0.001; Fig. 2a–g). Therefore, we selected age, BMI, sex, metastatic cancer, coagulopathy, vasoactive drug use, MEWS, lac concentration, RDW, NLR, INR, and osmolarity level for inclusion in the initial multivariate logistic regression analysis. The present selection strategy is more convenient to use in the clinic than the selection strategy in which all variables based on the results of the univariate analyses in the development cohort are included. The multivariate logistic analyses identified age, metastatic cancer, coagulopathy, MEWS, lac concentration, RDW, NLR, INR, and osmolarity level as independent risk factors for 28-day mortality. The adjusted OR values with 95% CIs for these variables are presented in Table 7. Furthermore, we evaluated the potential multicollinearity of the model above based on the VIF. The VIFs for the RDW, age, and osmolarity level in the prediction model for 28-day mortality were 12.39, 12.23, and 12.34, respectively, thus indicating the multicollinearity of the initial predictive model. To acquire an ideal model, we removed the RDW and age due to multicollinearity as well as coagulopathy given that the INR can simply reflect abnormal coagulation. Finally, we selected metastatic cancer, MEWS, lac concentration, NLR, INR, and osmolarity level for multivariate logistic regression analysis again to build a simplified model. The adjusted ORs together with the 95% CIs and VIF values for the simplified predictive model for 28-day mortality are listed in Table 7.

**Table 6:** Univariate analyses of factors associated with 28-day ICU mortality rate in development cohort.

	Unadjusted OR	95% CI	*p*		Unadjusted OR	95% CI	*p*
**Demographic data**				**Risk scores**			
Age (years)	1.033	1.031–1.035	< 0.001	MEWS	1.306	1.289–1.323	< 0.001
Body mass index	0.995	0.993–0.997	< 0.001				
Sex	0.880	0.823–0.940	< 0.001				
**Chronic medical conditions or comorbidities**				**Laboratory parameters**			
Heart and great vessel disease				Blood routine examination			
Congestive heart failure	1.526	1.424–1.635	< 0.001	White blood cell counts (× 10^9^/L)	1.074	1.065–1.082	< 0.001
Cardiac arrhythmias	1.520	1.419–1.627	< 0.001	Neutrophil to lymphocyte ratio	1.069	1.062–1.075	< 0.001
Pulmonary circulation disease	1.176	1.047–1.322	0.007	Hemoglobin (g/L)	0.889	0.865–0.914	< 0.001
Valvular disease	0.846	0.769–0.931	0.001	Red cell distribution width (%)	1.396	1.366–1.426	< 0.001
Hypertension	0.872	0.816–0.931	< 0.001	Platelets (× 10^12^/L)	1.000	1.000–1.001	0.429
Use of vasoactive drug (− 48 to 48 h)	1.812	1.680–1.954	< 0.001	Liver function test			
Digestive system diseases				Total bilirubin (mg/dL)	1.222	1.186–1.259	< 0.001
Liver disease	1.675	1.510–1.857	< 0.001	Aspartate transaminase (U/L)	1.001	1.001–1.001	< 0.001
Renal failure	1.309	1.204–1.424	< 0.001	Alanine transaminase (U/L)	1.001	1.001–1.001	< 0.001
Endocrine system diseases				Coagulation function			
Diabetes	0.924	0.858–0.995	0.037	International normalized ratio	1.198	1.171–1.226	< 0.001
Hypothyroidism	1.132	1.021–1.256	0.025	Prothrombin time (s)	1.081	1.073–1.090	< 0.001
Neurological and psychiatric diseases				Activated partial thrombo-plastin time (s)	1.009	1.007–1.010	< 0.001
Paralysis	1.224	1.038–1.444	0.018	Kidney function			
Other neurological disease	1.301	1.185–1.428	< 0.001	Blood urea nitrogen (mg/dL)	1.031	1.029–1.034	< 0.001
Psychoses	0.592	0.482–0.728	< 0.001	Serum creatinine (mg/dL)	1.580	1.509–1.655	< 0.001
Depression	0.602	0.525–0.690	< 0.001	Blood gas analysis			
Tumor				PH	0.051	0.032–0.082	< 0.001
Solid tumor	1.299	1.132–1.491	< 0.001	PaO_2_ (mmHg)	0.998	0.998–0.998	< 0.001
Metastatic cancer	2.833	2.548–3.150	< 0.001	PaCO_2_ (mmHg)	0.992	0.988–0.996	< 0.001
Lymphoma	1.720	1.412–2.095	< 0.001	SO_2_ (%)	0.983	0.977–0.990	0.002
Hematological diseases				PaO_2_/FiO_2_	0.998	0.998–0.999	0.007
Coagulopathy	2.100	1.930–2.293	< 0.001	Lactate (mmol/L)	1.302	1.278–1.326	< 0.001
Alcohol abuse	0.782	0.683–0.896	< 0.001	Electrolyte			
Drug abuse	0.498	0.390–0.637	< 0.001	Sodium (mmol/L)	1.060	1.048–1.072	< 0.001
				Osmolarity	1.053	1.050–1.056	< 0.001
				≥ 300 (mmoL/L)	2.059	1.887–2.247	< 0.001
				Blood glucose (mg/dL)	1.005	1.004–1.005	< 0.001

**Table 7:** Multivariate analyses and VIF assessment of factors associated with 28-day mortality rate in development cohort.

Variables	28-day mortality							
	Initial model				Simplified model			
	Adjusted OR	95% CI	*p*	VIF	Adjusted OR	95% CI	*p*	VIF
Age	1.038	1.036–1.041	< 0.001	12.23				
BMI			0.506					
Gender			0.309					
Metastatic cancer	2.641	2.320–3.007	< 0.001	1.12	2.791	2.474–3.150	< 0.001	1.10
Coagulopathy	1.575	1.416–1.751	< 0.001	1.20				
Use of vasoactive drug			0.113					
MEWS scores	1.240	1.220–1.259	< 0.001	6.47	1.223	1.205–1.241	< 0.001	6.20
NLR	1.034	1.026–1.041	< 0.001	4.87	1.045	1.038–1.053	< 0.001	4.49
RDW level	1.512	1.377–1.660	< 0.001	12.39				
INR	0.898	0.869–0.929	< 0.001	6.56	0.937	0.910–0.968	< 0.001	6.31
Lac	1.253	1.212–1.290	< 0.001	7.34	1.230	1.197–1.263	< 0.001	7.27
Osmolarity level	1.461	1.322–1.615	< 0.001	12.34	1.669	1.517–1.836	< 0.001	7.68
Mean VIF = 7.21				Mean VIF = 5.51, H/L C-statistic = 5.64 (*p* = 0.688)				

**Figure 3:**
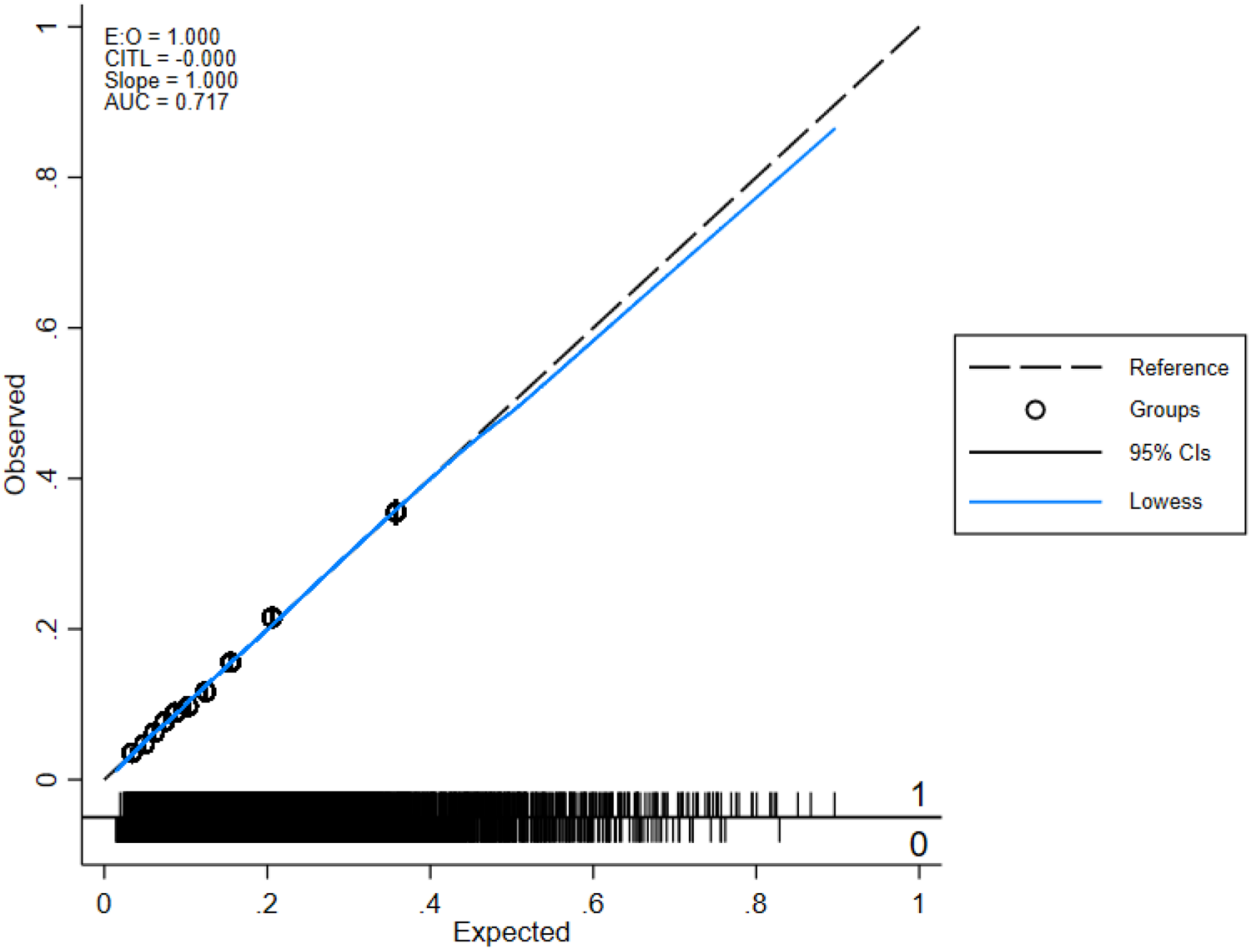
Calibration plot of observed versus expected probabilities for assessment of the predictive performance of the mionl-MEWS model in the development cohort.

**Figure 4:**
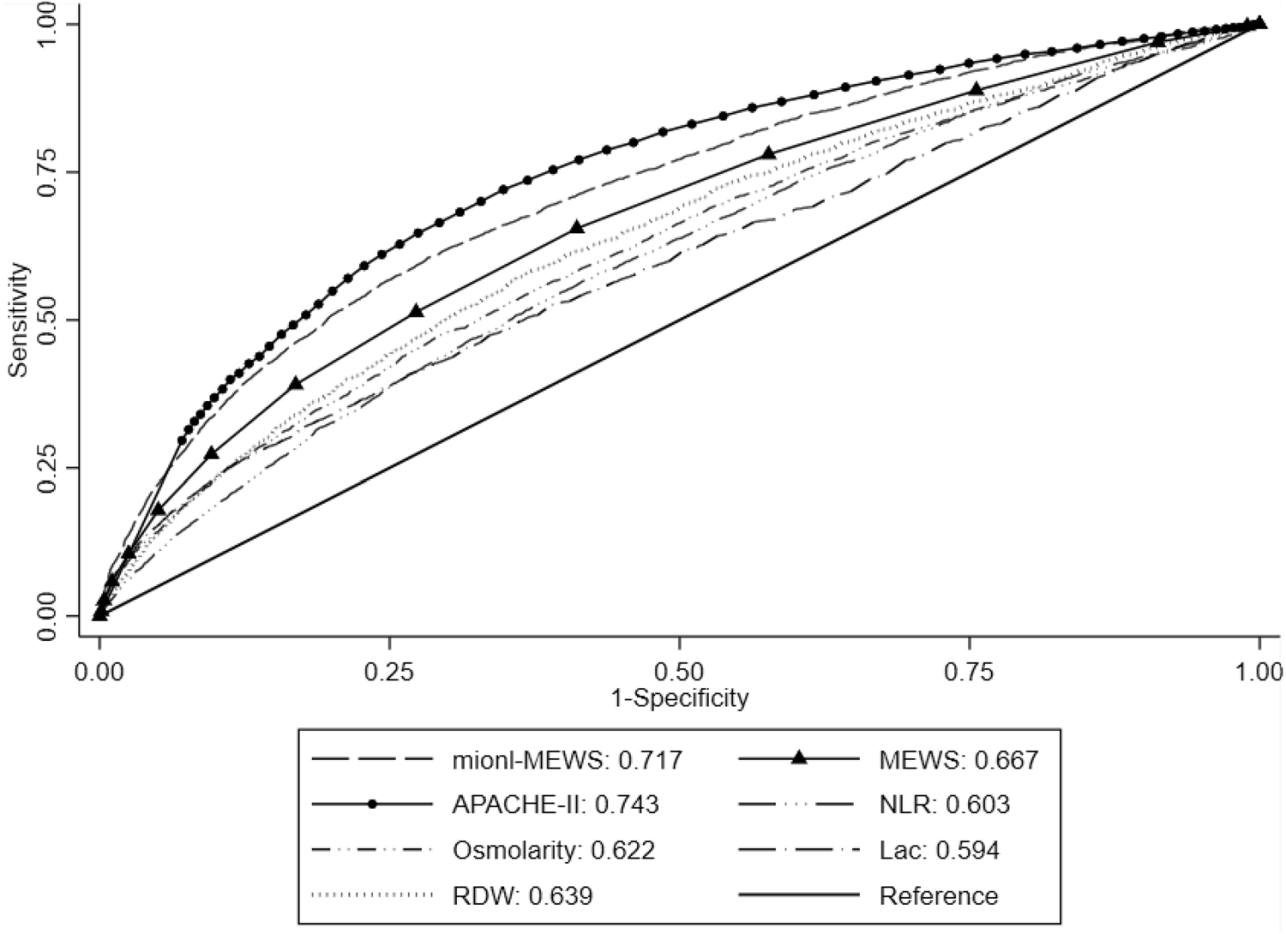
Predictive performance of the mionl-MEWS, APACHE-II, MEWS, neutrophil-to-lymphocyte ratio (NLR), red cell distribution width (RDW), lactate (lac) concentration, and osmolarity level for 28-day mortality in critically ill patients in the development cohort.

Considering that this predictive model was constructed based on the MEWS, NLR, lac concentration, INR, osmolarity level, and presence of metastatic cancer, the model was named the “mionl-MEWS” model. The AUROC for 28-day mortality using the mionl-MEWS for critically ill patients was 0.717 (95% CI 0.708–0.726, *p* < 0.001). The calculated H/L C-statistic was equal to 11.27 (*p* = 0.187), and the calibration plot of the observed versus expected probabilities for assessment of model performance is displayed in Fig. 3. The AUROC values for the APACHE-II, MEWS, RDW, NLR, lac concentration, and osmolarity were 0.743, 0.667, 0.639, 0.603, 0.594, and 0.622, respectively (Table 8). Statistical differences were detected among these AUROC (*p* < 0.001; Fig. 4). The Brier scores, which indicate model accuracy for measuring prediction at an individual level, were 0.097 (*p* = 0.575) for the mionl-MEWS, 0.102 (*p* = 0.673) for the APACHE-II, 0.108 (*p* = 0.575) for the MEWS, 0.110 (*p* = 0.492) for RDW, 0.109 (*p* = 0.574) for lac concentration, 0.112 (*p* = 0.507) for the NLR, and 0.111 (*p* = 0.671) for osmolarity (Table 8).

**Table 8:** Performance of mionl-MEWS, APACHE-II, MEWS, RDW, NLR, and lac for predicting 28 day-mortality in critically ill patients in the development and validation cohorts.

Performance	mionl-MEWS*	APACHE-II*	MEWS*	RDW*	NLR*	Lac*	Osmolarity*
**Predictive efficiency for 28-day mortality in development cohort**							
AUROC	0.717 (0.708–0.726)	0.743 (0.734–0.751)	0.667 (0.658–0.677)	0.639 (0.629–0.649)	0.603 (0.593–0.613)	0.594 (0.583–0.604)	0.622 (0.612–0.632)
Brier score	0.097 (*p* = 0.575)	0.102 (*p* = 0.673)	0.108 (*p* = 0.575)	0.110 (*p* = 0.492)	0.112 (*p* = 0.507)	0.109 (*p* = 0.574)	0.111 (*p* = 0.671)
Performance	mionl-MEWS*^#^	APACHE-II	MEWS*	RDW*	NLR*	Lac*	Osmolarity*
**Predictive efficiency for 28-day mortality in validation cohort**							
AUROC	0.908 (0.883–0.933)	0.884 (0.853–0.915)	0.877 (0.846–0909)	0.712 (0.662–0.761)	0.630 (0.577–0.682)	0.729 (0.682–0.775)	0.751 (0.705–0.797)
Brier score	0.122 (*p* = 0.540)	0.102 (*p* = 0.287)	0.111 (*p* = 0.538)	0.138 (*p* = 0.326)	0.157 (*p* = 0.421)	0.138 (*p* = 0.512)	0.163 (*p* = 0.890)

*mionl-MEWS versus APACHE-II or MEWS or RDW or NLR or Lac or Osmolarity (p < 0.001).

^#^mionl-MEWS versus APACHE-II (*p* = 0.120), *mionl-MEWS versus MEWS or RDW or NLR or Lac or Osmolarity (*p* < 0.001).

### Internal validation of the mionl-MEWS score

**Figure 5:**
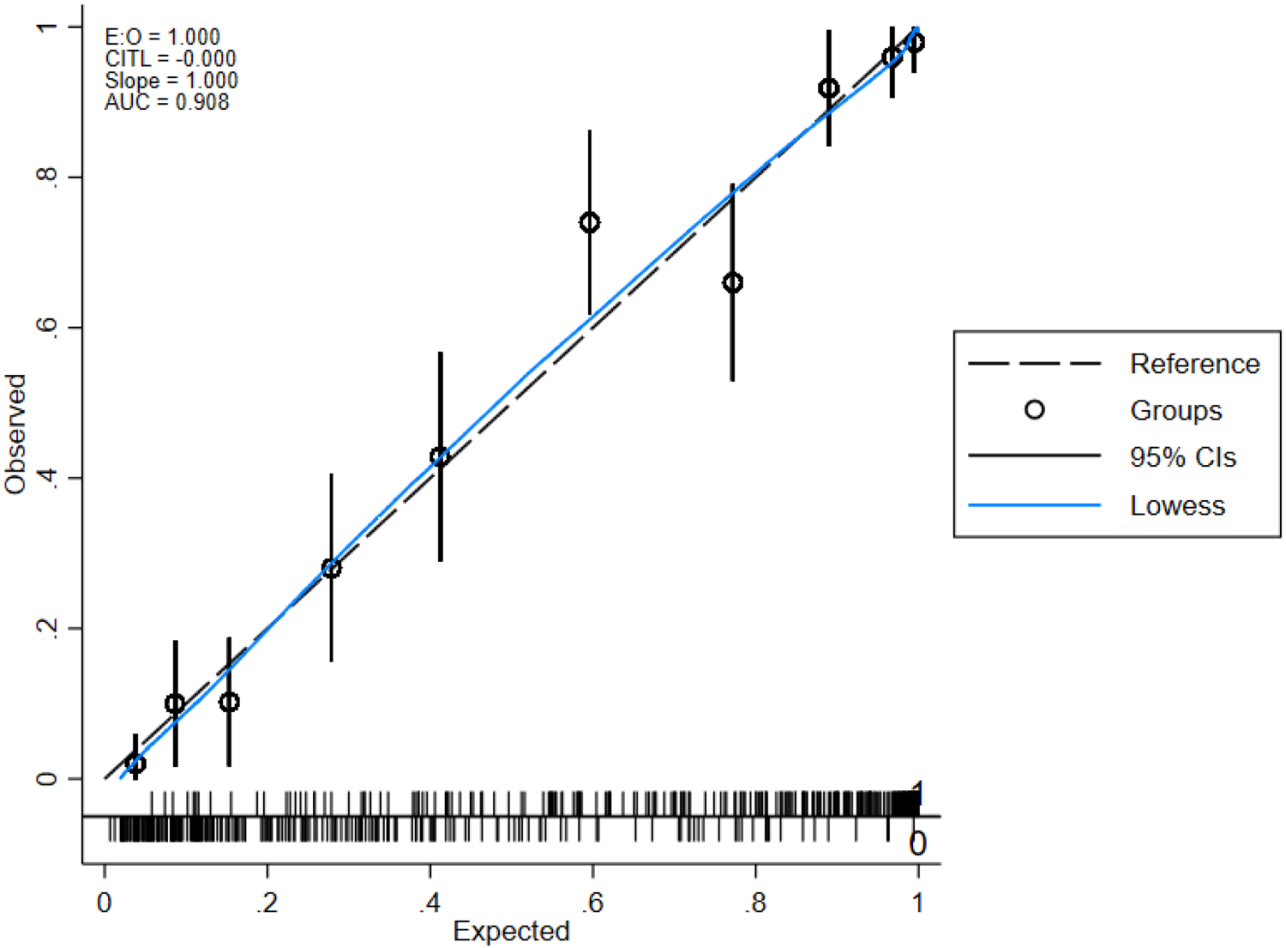
Calibration plot of observed versus expected probabilities for assessment of the predictive performance of the mionl-MEWS model in the development cohort.

**Figure 6:**
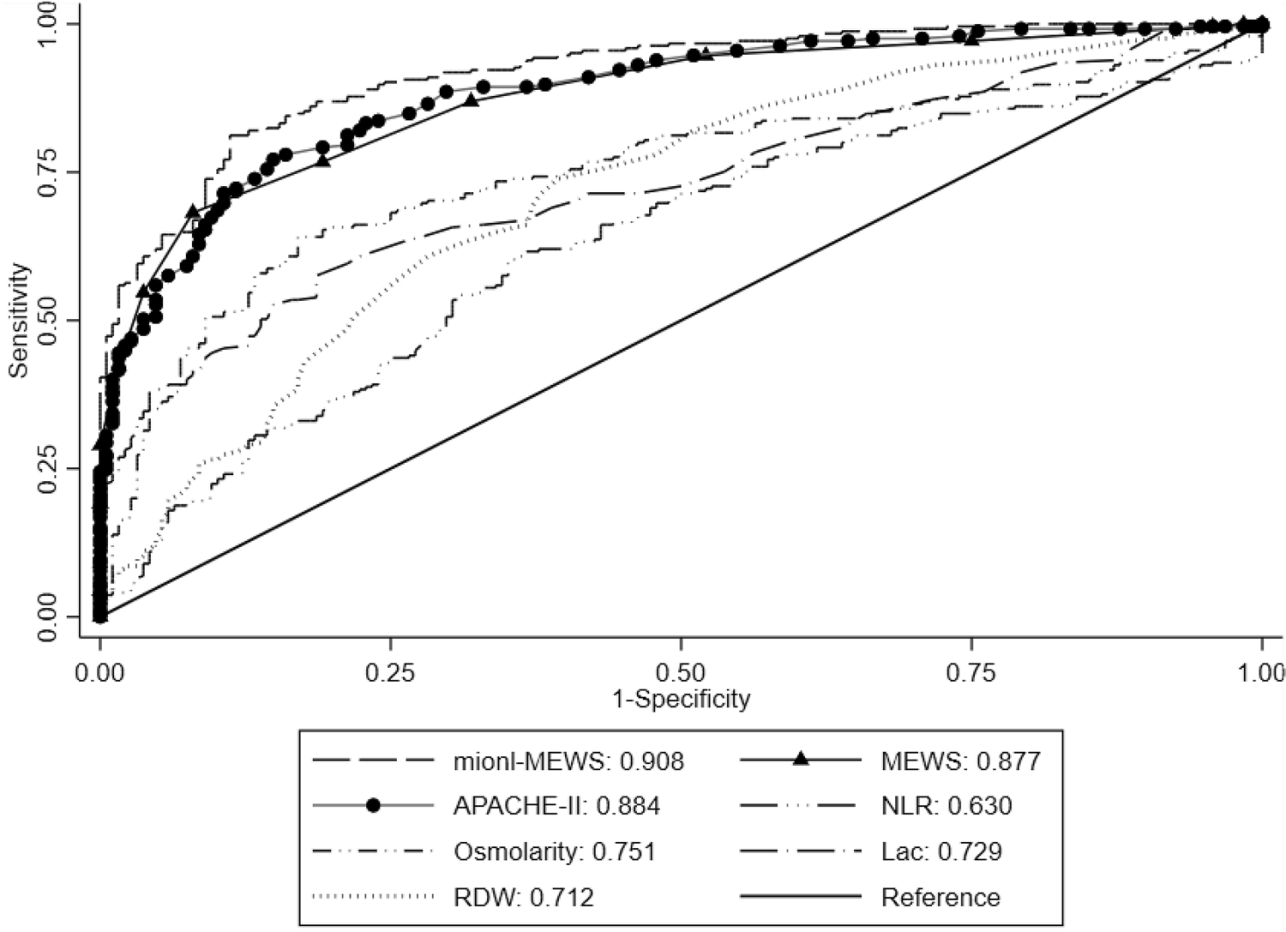
Predictive performance of the mionl-MEWS, APACHE-II, MEWS, neutrophil-to-lymphocyte ratio (NLR), red cell distribution width (RDW), lactate (lac) concentration, and osmolarity level for 28-day mortality in critically ill patients in the validation cohort.

**Figure 7:**
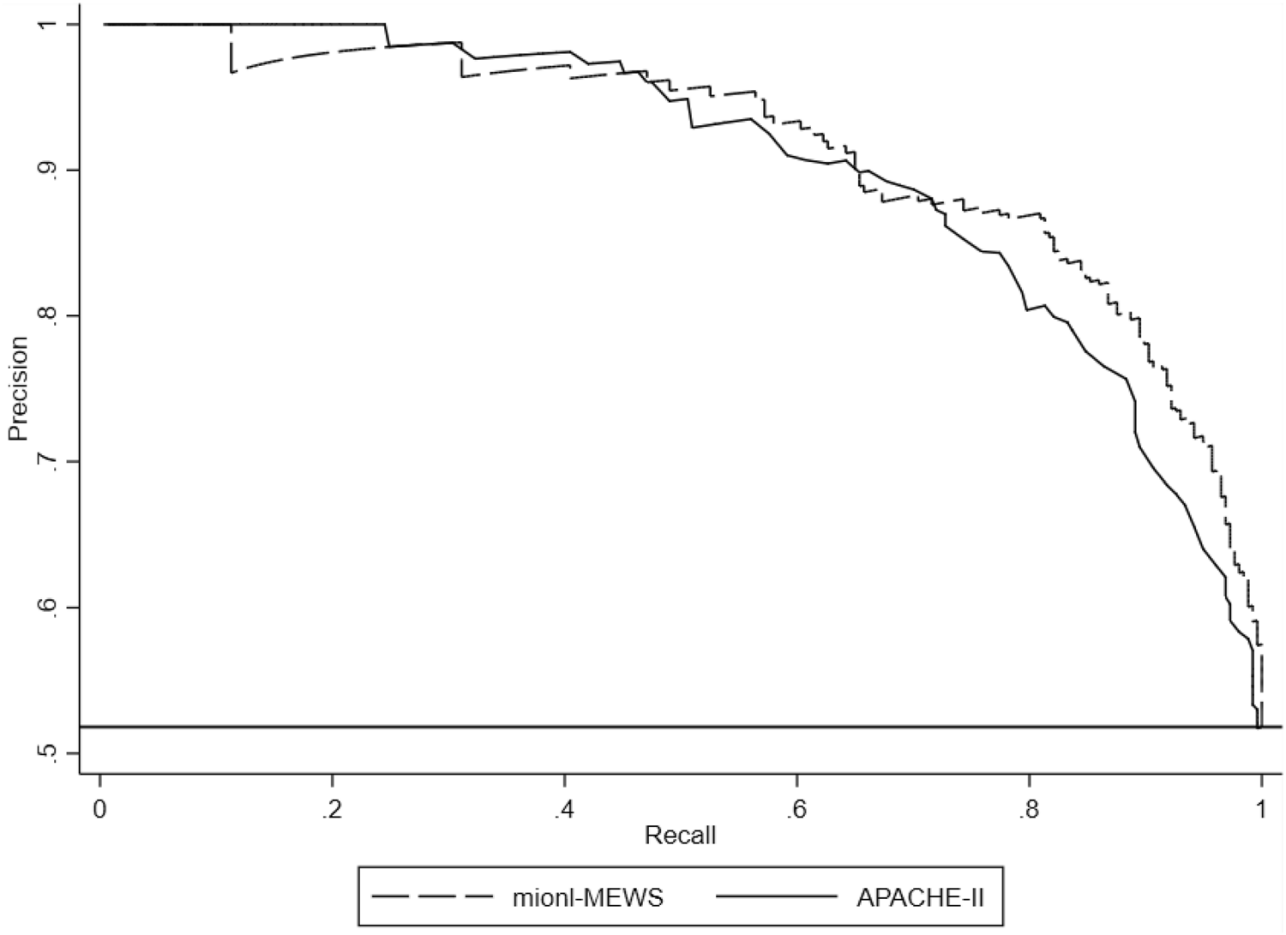
Comparison of precision-recall area under the curves (PR-AUCs) between the mionl-MEWS and APACHE-II in the validation cohort.

Next, we internally validated the mionl-MEWS model in the validation group. All VIF values for the mionl-MEWS model are listed in Table 9. The H/L C-statistic in the validation group was equal to 12.33 (*p* = 0.518), and the calibration plot is displayed in Fig. 5. The AUROC for the mionl-MEWS model for predicting 28-day mortality among ICU patients demonstrated good discriminative power in the validation group (0.908, 95% CI 0.883–0.933, *p* < 0.001). The AUROC values for the APACHE-II, MEWS, RDW, NLR, lac concentration, and osmolarity in the validation group were 0.883 (0.853–0.915), 0.877 (0.846–0909), 0.712 (0.662–0.761), 0.630 (0.577–0.682), 0.729 (0.682–0.775), and 0.751 (0.705–0.797), respectively (Table 8). Similarly, statistical differences were also detected among these AUROC values (*p* < 0.001). Although the AUROC for the mionl-MEWS appeared to be greater than that for the APACHE-II, the difference was not found to be significant (*p* = 0.120; Fig. 6). The PR-AUCs for the mionl-MEWS and APACHE-II were 0.907 and 0.899, respectively (Fig. 7). The Brier scores were as follows: mionl-MEWS, 0.122 (*p* = 0.540); APACHE-II, 0.102 (*p* = 0.287); MEWS, 0.111 (*p* = 0.538); RDW, 0.138 (*p* = 0.326); lac, 0.138 (*p* = 0.512); NLR, 0.157 (*p* = 0.421); and osmolarity, 0.163 (*p* = 0.890) (Table 8). These results indicate that the mionl-MEWS had good predictive ability with great calibration abilities. Importantly, the mionl-MEWS was not found to be inferior to the APACHE-II and was shown to be superior to other risk scores in the validation group.

**Table 9:** VIF assessment of factors associated with 28-day mortality rate in validation cohort.

Variables	VIF
Metastatic cancer	1.10
MEWS	7.30
NLR	1.85
INR	1.42
Lac	2.78
Osmolarity level	6.08
Mean VIF = 3.42, H/L C-statistic = 12.33 (*p* = 0.518)

### k-Fold cross validation of the mionl-MEWS score

**Figure 8:**
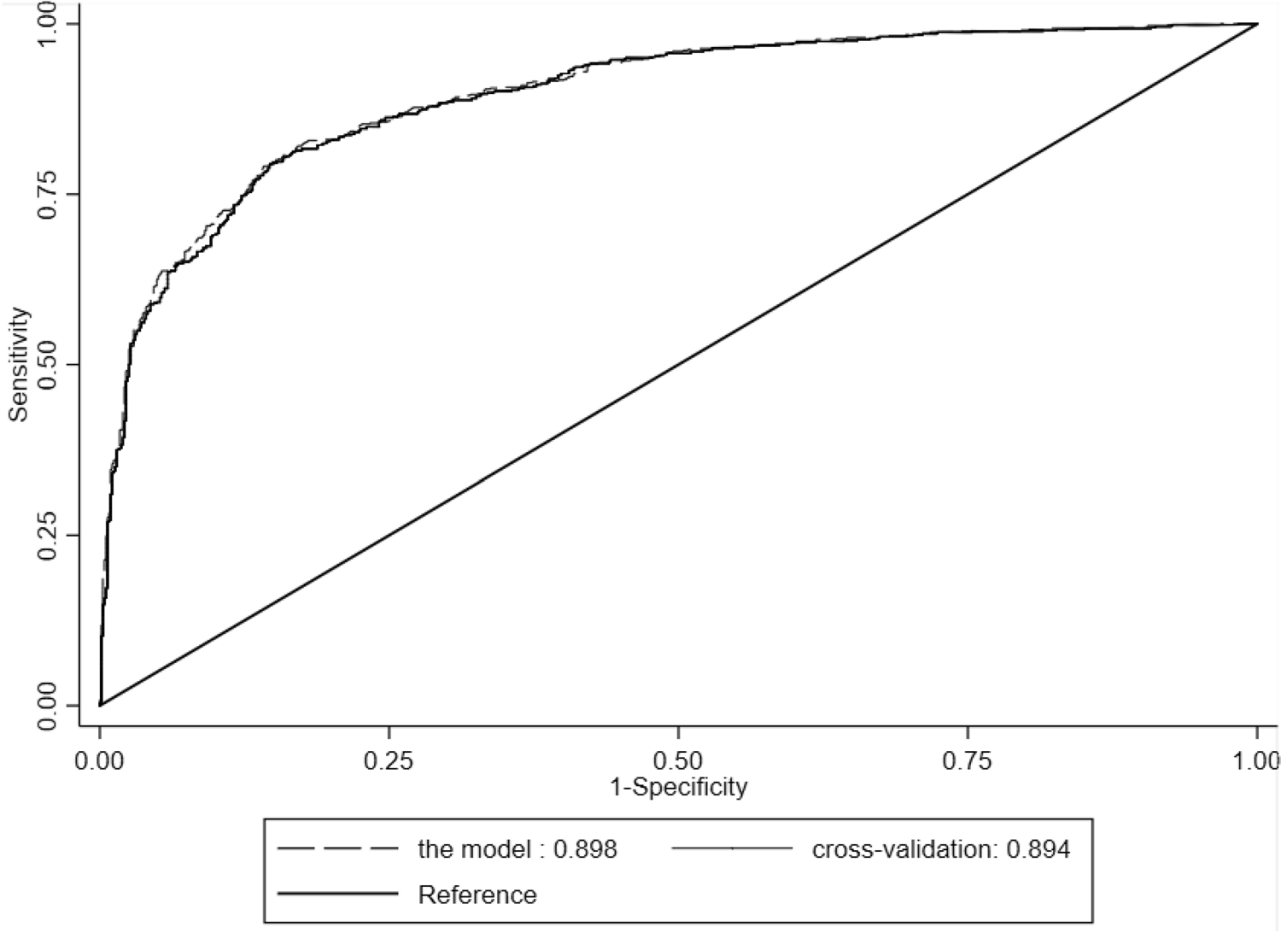
Comparison of the area under the receiver operating characteristic curve (AUROC) values between the mionl-MEWS and cross validation.

To further illustrate the robustness of the developed mionl-MEWS model, we used repetitive randomization and k-fold cross validation (k = 5) to analyze the total of 51,121 patients. The AUROC for our model was 0.898 and that with k-fold cross-validation was 0.895 (Fig. 8). Under k-fold cross validation, the PPV and NPV were similar between our model and k-fold cross-validation (PPV 0.842 vs. 0.847 and NPV 0.805 vs. 0.810, respectively).

### Nomogram for the mionl-MEWS score

**Figure 9:**
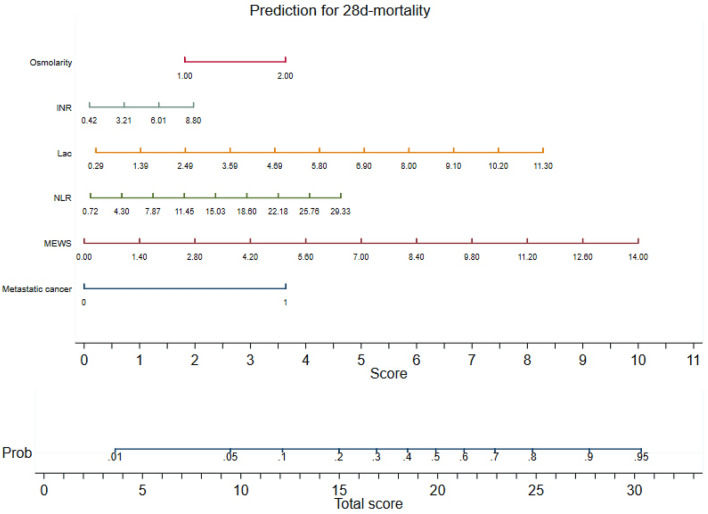
Nomogram for the mionl-MEWS model. On the nomogram, an individual patient’s predicted mortality risk according to the mionl-MEWS model is located on each variable axis, and a line is drawn upward to determine the corresponding score for each variable state. The sum of these numbers indicates the total score, and a line is drawn to the probability axis to determine the likelihood of 28-day mortality (*INR* international normalized ratio, *Lac* lactate, *MEWS* Modified Early Warning Score, *NLR* neutrophil-to-lymphocyte ratio).

Because the AUROC value provides limited information regarding how a prediction score works in clinical practice, a nomogram is needed to visualize the prognostic model for clinicians, and this graph is useful in resource-limited settings such as those without statistical software or electronic calculators. We translated the model with integrated independent factors into a nomogram using Stata statistical software. The prognostic nomogram derived from the mionl-MEWS score for clinical application is shown in Fig. 9.

## Discussion

To the best of our knowledge, this retrospective study is the first to propose a simple prognostic model (mionl-MEWS) combining metastatic cancer, MEWS, lac, NLR, INR, and osmolarity level for the prediction of 28-day mortality in critically ill patients with internal validation. Based on the AUROC and PR-AUC values, the predictive efficacy of the mionl-MEWS for 28-day mortality in critically ill patients was superior to that of the traditional MEWS, NLR, RDW, lac, or osmolarity alone. Hence, the mionl-MEWS could be used to assist with clinical decision-making in the management of ICU patients.

Considering the likelihood of long in-hospital stays and high medical resource consumption, early identification of mortality risk using prognostic scoring systems is important for timely and effective management and intervention in critically ill patients in the ICU. In addition, patterns of ICU admissions have changed due to advances in the treatment of solid malignancies with immunotherapy and targeted therapies. For example, the proportion of patients with metastatic diseases increased from 48.6% in 2007–2008 to 60.2% in 2017–2018 in France^18^. Although many scoring systems for critical illness have been proposed to translate the complexity of patients’ conditions into a single measure based on quantitative survival probabilities in current clinical practice, the drawbacks and flaws of these individual systems cannot be ignored. For instance, some assessment tools require many blood tests and/or scoring items, which can be time-consuming and lead to delayed interventions and/or a high financial burden for patients. Thus, fast, convenient, and inexpensive evaluation tools are needed in clinical practice.

Our study retrospectively collected variables that could predict the 28-day mortality in critically ill patients. These variables, such as the MEWS, lac, NLR, INR, etc., were chosen from the literature and used in previous ICU risk assessment models. In our study, we demonstrated that compared with survivors, nonsurvivors tended to be older; male; have a higher incidence of metastatic cancer, coagulopathy, and vasopressor drug use within 48 h; have a lower BMI; and have higher MEWS, RDW, NLR, lac, INR, and osmolarity values, indicating that these factors might serve as potential prognostic markers in critically ill patients. Next, we investigated the factors that independently predicted 28-day mortality in critically ill patients. Our initial multivariate logistic regression analysis also showed that age, metastatic cancer, coagulopathy, MEWS, lac concentration, NLR, RDW, INR, and osmolarity level were independent predictors for 28-day mortality. Unfortunately, multicollinearity was detected among age, RDW, and osmolarity level. However, a series of studies have demonstrated that RDW has predictive value for mortality in patients with heart failure, septic shock, acute respiratory distress syndrome, etc.^14,19,20^. In addition, age ≥ 80 years was shown to be associated with higher ICU and hospital death compared with younger ages^21^. In our study, RDW and age also showed a correlation with the mortality of critically ill patients (OR 1.512; 95% CI 1.377–1.660; OR 1.038; 95% CI 1.036–1.041, *p* < 0.001, respectively). Nevertheless, in a previous cohort study of 8089 individuals analyzing the effect of age and RDW, the age-dependency of RDW seemed to be a universal biological feature^22^. Therefore, we removed age and RDW from our model to avoid multicollinearity in subsequent modeling.

Among the three underlying disease variables, metastatic cancer was previously shown to be an important predictor of a high 30-day mortality in the ICU^23^ along with mechanical ventilation and vasopressor use^24^. In the present study, the OR value for metastatic cancer as a predictor of 28-day mortality was 2.791 (95% CI 2.474–3.150; *p* < 0.001), which is similar to that reported by Barth et al. for the outcome of patients with metastatic lung cancer admitted to the ICU (OR 4.22 [1.4–12.4]; *p* = 0.008)^24^. Therefore, tumor metastasis should be considered in the decision-making process in the ICU. Coagulopathy also is a common cause for a poor prognosis in critically ill patients in the ICU, and its severity has been shown to predict hospital mortality standardized by INR^25^. Therefore, we only selected INR for inclusion in the final model analysis. Finally, vasopressors are commonly administered to ICU patients with hypotension to raise patients’ blood pressure^26^. Decision-making regarding the timing of vasopressor initiation as well as balancing the risks and benefits of vasopressor use remains challenging. In the dataset used in our study, the proportion of patients who required treatment with a vasopressor within 48 h was significantly higher in the nonsurvivor group than in the survivor group (27.8% vs. 17.52% *p* < 0.001). Interestingly, vasopressor use was not found to be an influencing factor in our multiple regression analysis though. In a cohort study regarding the mortality of septic shock patients, only a weak correlation between the timing of vasopressor initiation and hospital mortality was found (adjusted OR 1.02, 95% CI 1.01–1.03, *p* < 0.001)^27^. These results also indirectly corroborate the finding in the present study that the timing of vasopressor initiation might not be associated with 28-day mortality in the ICU.

Among the indexes, MEWS was developed as a practical tool that can rapidly and effectively estimate clinical death risk using only five simple and basic physiological parameters without increasing the economic burden, since these parameters can be acquired from patient’s electronic medical records automatically. In a previous observational study, Moon et al. found that the introduction of MEWS charts significantly reduced the number of in-hospital cardiac arrest calls (2% vs. 3%; *p* = 0.004) and in-hospital mortality rates (42% vs. 52%; *p* = 0.05)^28^. In addition, in a study predicting the 28-day mortality rate of ICU patients with severe septic shock, the MEWS was associated with the 28-day mortality rate (OR 1.462; 95% CI 1.122–1.905; *p* = 0.005)^29^, which was consistent with the finding in our study (OR 1.250; 95% CI 1.232–1.269; *p* < 0.001). However, another study found that the MEWS had limited ability to estimate sudden disease aggravation in patients, such as the occurrence of cardiac shock^30^. Therefore, the predictive value of the MEWS alone for the mortality rate in critically ill patients required further investigation.

Sepsis is well-recognized major health problem in the ICU globally. One study found that the proportion of ICU patients with ICU-acquired sepsis was 24.4% and that the mortality of hospitalized sepsis patients was very high at 25–30%^31^. Whether patients had sepsis was an important factor affecting the mortality of ICU patients. NLR, as an immune-related biomarker, was shown to serve as a convenient prognostic marker in sepsis patients. In their study predicting 28-day mortality in sepsis patients, Liu et al. reported that the NLR was associated with the 28-day mortality rate (OR 1.340; 95% CI 1.253–1.434; *p* < 0.001)^32^. However, in the present study, the OR value for the NLR was only 1.045 (95% CI 1.038–1.053; *p* < 0.001). This consistency might be due to differences in the study populations, as Liu et al. only selected patients with sepsis, and the present population was based on all ICU patients, not only those with sepsis. Previously, the lac concentration has been associated with mortality in different groups of critically ill patients, such as those with cardiogenic, hypovolemic, or septic shock. Relative hyperlactatemia (1.36–2.00 mmol/L) within the first 24 h of ICU admission was reported to be an independent predictor for in-hospital and ICU mortality in critically ill patients^16^. In addition, osmolarity with a threshold of 300 mmol/L was shown to be associated with increased mortality in critically ill patients with cardiac, cerebral, vascular, or gastrointestinal diagnoses at admission^33^, and these findings are consistent with those of our study (OR 1.669; 95% CI 1.517–1.836; *p* < 0.001).

Due to the complexity and heterogeneity in disease among critically ill patients, combination of different indexes can more accurately reflect the prognosis of ICU patients than any single index^34^. Thus, we included metastatic cancer, MEWS, lac concentration, NLR, INR, and osmolarity level in our model to predict 28-day mortality. Using the ROC curves to evaluate the 28-day mortality of critically ill patients, a higher AUROC values in the development cohort (0.717) and the validation cohort (0.908) were found upon combination of these six parameters as a composite index compared with each parameter separately. Notably, the mionl-MEWS had the greatest AUROC value, superior to those of the MEWS, RDW, osmolarity, NLR, and lac alone, indicating that the mionl-MEWS can provide a more comprehensive reflection of each patient’s condition from six dimensions, including metastatic cancer for the distribution characteristics of ICU patients, MEWS for patients’ general condition, lac concentration for microcirculation, NLR for sepsis, INR for coagulopathy, and osmolarity for the internal environment.

Furthermore, we used the Brier score to assess the accuracy of our developed model. Among the evaluated indexes, the mionl-MEWS had the smallest Brier score in the development cohort and the third lowest score in the validation cohort, indicating that the mionl-MEWS offered good accuracy for prediction at an individual level. Additionally, we calculated the H/L C-statistic to assess consistent agreement between the observed ICU mortality and the actual ICU mortality. The mionl-MEWS showed adequate calibration, suggesting the assignment of the correct probability at all levels of predicted risk. Finally, the mionl-MEWS model provided stable evaluation with excellent calibration in the validation group (AUROC: 0.908 and PR-AUC: 0.907).

Our study has some strengths. First, to our knowledge, this study is the first to demonstrate enhanced prognostic ability for 28-day mortality in ICU patients via the combination of metastatic cancer, MEWS, lac concentration, NLR, INR, and osmolarity level. Second, the sample size in our study was relatively large, which reduced selection bias. Furthermore, we applied different probability models to evaluate the mionl-MEWS model in order to ensure the scientific nature and credibility of the results. Third, the parameters included in the mionl-MEWS model are objective and easily accessible among laboratory parameters that are widely available to clinicians. Fourth, the constructed nomogram makes 28-day mortality prediction easy and rapid in clinical practice.

Nevertheless, it is important to recognize the limitations of our study. Our data were collected retrospectively from the MIMIC-III database, and because this was a single-center retrospective study, it might be difficult to extend the findings to other hospitals. External validation in cohorts from other countries is needed to generalize our findings. Additionally, due to incomplete data collection and inaccurate data elements from the MIMIC-III database, the potential for bias cannot be excluded.

## Conclusion

In the present study, we developed a prediction model, the mionl-MEWS, for the 28-day mortality of critically ill patients admitted to the ICU, demonstrated internal validation, and ensured the included clinical variables can be easily obtained in resource-limited settings. Our results showed that the mionl-MEWS offered higher predictive value for the 28-day mortality of critically ill patients compared with other scoring variables and/or systems. However, additional research is required to demonstrated whether the mionl-MEWS can be applied widely and extensively.

## Data Availability

All relevant data are freely available to any scientist wishing to use them for noncommercial purposes, after users first complete a mandatory training, without breaching participant confidentiality. The datasets generated and/or analyzed during the current study are available in the StataData1 repository, https://github.com/WX271/StataData1.

## Abbreviations

APACHE: Acute Physiology and Chronic Health Evaluation
AUROC: Area under the receiver operating characteristic curve
BMI: Body mass index
CI: Confidence interval
H/L C-Statistic: Hosmer–Lemeshow C-statistic
ICU: Intensive care unit
INR: International normalized ratio
lac: Lactate
LMICs: Low- and middle-income countries
MEWS: Modified Early Warning Score
MIMIC-III: Medical Information Mart for Intensive Care III
NLR: Neutrophil-to-lymphocyte ratio
OR: Odds ratio
PR-AUC: Precision-recall area under the curve
RDW: Red cell distribution width
VIF: Variance inflation factor
